# The resveratrol-enriched rice DJ526 boosts motor coordination and physical strength

**DOI:** 10.1038/srep23958

**Published:** 2016-04-05

**Authors:** Hea-Jong Chung, Satya Priya Sharma, Hyeon-Jin Kim, So-Hyeon Baek, Seong-Tshool Hong

**Affiliations:** 1 Department of Biomedical Sciences and Institute for Medical Science, Chonbuk National University Medical School, Jeonju, Chonbuk 54896, South Korea; 2 JINIS BDRD institute, JINIS Biopharmaceuticals Co., 948-9 Dunsan, Bongdong, Wanju, Chonbuk 55321, South Korea; 3 Department of Well-being Resources, Sunchon National University, Suncheon, Jeonnam 57922, South Korea

## Abstract

The main objective of current genetic modifications in crops is to boost agricultural production or to develop GM crops with an improved nutrient profile by introducing a new trait to the plants. A GM crop surpassing the ability of the introduced genetic characteristics has not been developed yet. Here, we show that the resveratrol-enriched rice DJ526, a GM crop, has unexpectedly high beneficial health effects surpassing the introduced genetic characteristic of resveratrol synthetic ability. The synergistic effect of its innate and transgenic properties not only ameliorates age-related deterioration but also boosts motor coordination and physical strength during the aging process. The gene expression profiling analyses by DNA chip showed that the gene expression pattern of mice fed resveratrol-enriched rice DJ526 was very different from mice fed either resveratrol or Dongjin rice alone, respectively, modifying expression of genes related to aging regulation, cell differentiation, extracellular matrix, neurogenesis, or secretion.

Starvation has been an eminent threat to humankind until the green revolution, and thus, the prime objective of agricultural science was to increase agricultural production. The green revolution, occurring between the 1940s and the late 1960s, increased agricultural production dramatically^1^,^2^. Although the problem of shortage of agricultural production in the world has been mostly solved by the green revolution, the increase of agricultural production still remains a prime objective in agricultural science^3^. Therefore, it is not surprising that the main aim of genetic modifications in crops is to introduce a new trait to the plants for increasing agricultural production at a low cost^4^. Examples of genetically modified (GM) crops to boost agricultural production include resistance to pests, diseases, or environmental conditions, reduction of spoilage, or resistance to herbicides^5^,^6^. Additionally, there have been attempts to develop GM crops with an improved nutrient profile such as golden rice^7^.

Mammalian bodies, including human beings, are extremely complicated with numerous metabolic and signaling pathways. Because of the complexity of numerous pathways, nutrients or chemicals co-ingested into mammalian bodies interact in one of three ways–addictive, synergistic, or antagonistic. In the addictive effect, ingested components do not affect each other, so the biological effect is independent each other. The genetic modification of a crop to improve the nutrient profile could be an example of an addictive effect in which the original and fortified nutrients of the GM crop work independently^8^,^9^. On the other hand, in a synergistic effect, ingested components work together to produce an enhanced result, sometimes producing unexpected, enhanced effects like new types of beneficial effects. Although nutrients or chemicals in mammalian bodies mostly work in an addictive manner, synergistic interactions are frequently observed. Considering the fact that the synergistic effect is much more beneficial than the addictive effect, engineering of the synergistic effect in GM crop development could create a functional crop to treat and/or prevent various diseases. However, there has not yet been an attempt to develop a GM crop with synergistic effects from innate and transgenic properties.

Previously, we created the resveratrol-enriched rice DJ526 by transferring the resveratrol biosynthesis gene, stilbene synthase, from the peanut *Arachis hypogaea* variety Palkwang into the rice *Oryza sativa* japonica variety Dongjin^10^,^11^. The resveratrol-enriched rice DJ526 accumulated 1.4–1.9 μg/g of resveratrol in its grain. Dongjin rice is rich in fiber and polyphenols that improve age-related diseases such as metabolic syndrome and obesity, among others^12^. Although Dongjin rice itself has an insignificant effect on metabolic syndrome and obesity, the genetically modified resveratrol-enriched rice DJ526 shows unexpectedly high efficacy for treating metabolic syndrome and obesity in animal studies^10^,^11^, with a pharmacological efficacy comparable to typical pharmaceutical drugs aimed to treat these diseases^10^,^11^. Considering the fact that the concentration of resveratrol in the resveratrol-enriched rice DJ526 was much lower than the efficacy level of resveratrol in typical mouse experiments showing any beneficial effect^13^,^14^,^15^, the pharmacological efficacy of the resveratrol-enriched rice DJ526 was suspected to be rooted in the synergistic effect of its innate and transgenic properties.

In this work, we proved how the innate trait of the resveratrol-enriched rice DJ526 acts synergistically with the transgenic trait of resveratrol in mice to confer unexpectedly high health benefits. The resveratrol-enriched rice DJ526 ameliorates age-related deteriorations in mice, as exhibited by a boost in motor coordination and physical strength during aging, as well as by detection from gene expresses profiling analyses of expressed genes involved in the anti-aging processes. This work suggests that functional crops with significant beneficial health effects could be generated if the synergistic interactions of transgenic properties with endogenous traits were considered during GM crop creations.

## Results

### Mice fed Dongjin rice or the resveratrol-enriched rice DJ526 maintained a healthy body weight with age-progression

**Figure 1:**
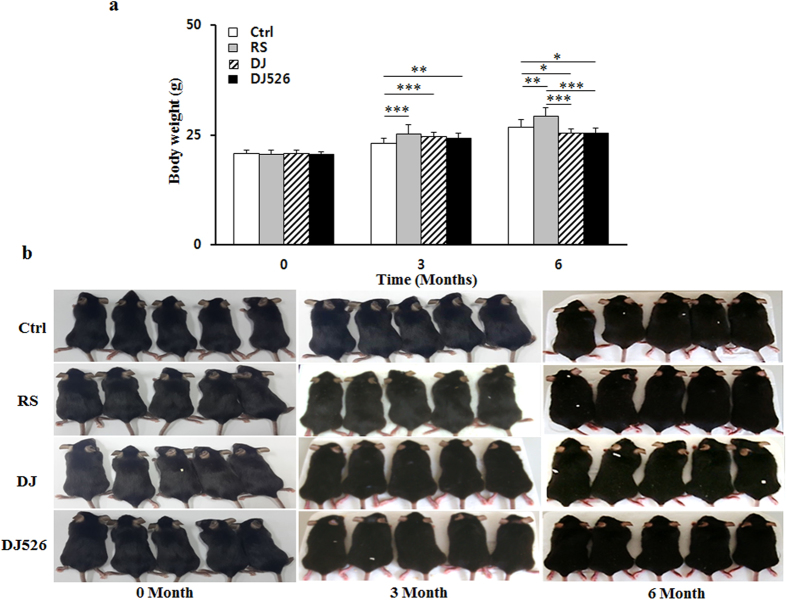
The effects of the resveratrol-enriched rice DJ526 on changes in body weight with age progression. (**a**) Changes in the body weight of four experimental groups mice at 0, 3 and 6 months of the experiment. The values represent the mean ± s.d. (n = 20). The control (Ctrl) mice fed a NFD in which the carbohydrate source was corn starch and sucrose; resveratrol (RS) mice fed a NFD in which the carbohydrate source was corn starch and sucrose except containing resveratrol; Dongjin (DJ) mice fed a NFD in which the corn starch and sucrose were replaced with Dongjin rice; DJ526 mice fed a NFD in which the corn starch and sucrose were replaced with the resveratrol-enriched rice DJ526. The resveratrol concentration in DJ526 and RS groups was 0.69 μg/g each, and not detected in Ctrl and DJ groups. Values in the figure with a superscripted letter indicate statistical significance as analyzed by an unpaired Student’s t-test; *p < 0.05, **p < 0.01, ***p < 0.001. (**b**) Images representing the physical status of experimental mice groups at 0, 3 and 6 months.

Our previous studies showed that the resveratrol-enriched rice DJ526 was effective in treating obesity and metabolic syndrome through a synergistic combination of the innate anti-obesity property of Dongjin rice and the lipid-lowering property of transgenic resveratrol^10^,^11^. However, those animal experiments were conducted in high calorie-induced conditions, limiting their interpretation as a therapeutic option for human use. To evaluate the efficacy of the resveratrol-enriched rice DJ526 as a daily consumable food source as well as its synergistic mechanism, we analyzed the effect of the resveratrol-enriched rice DJ526 during normal diet conditions. This was done by replacing the carbohydrate source of the non-fat normal formula diet with the resveratrol-enriched rice DJ526 (see Supplementary Table S1). The gross effect of the resveratrol-enriched rice DJ526 was observed by evaluating body weight and physical appearance of each mouse group with age progression. Daily recording of food consumptions showed that the food consumption rate of four experimental groups of mice in Fig. 1 was the same during the experimental period. As shown in Fig. 1a, the body weights of the DJ526 group (mice fed with the resveratrol-enriched rice DJ526) or the DJ group (mice fed with Dongjin rice) were maintained during age progression, while the control group (mice fed with non-fat normal formula diet) and the RS group (mice fed with non-fat normal formula diet containing resveratrol equivalent to that of the DJ526 group) slowly gained body weight during the experimental period. Because weight gain with age progression is one of the important symptoms of aging, these results are in accordance with the previous reported facts of general health beneficial efficacy of Dongjin rice^12^. However, despite the fact that both of Dongjin rice and the resveratrol-enriched rice DJ526 could affect beneficially an age-related deterioration, the general physical appearance of the DJ526 group, which was assessed by the coat color and shape, was healthier than that of the DJ group (Fig. 1b).

### Mice fed Dongjin rice or the resveratrol-enriched rice DJ526 maintained a healthy fat content with age progression

**Figure 2:**
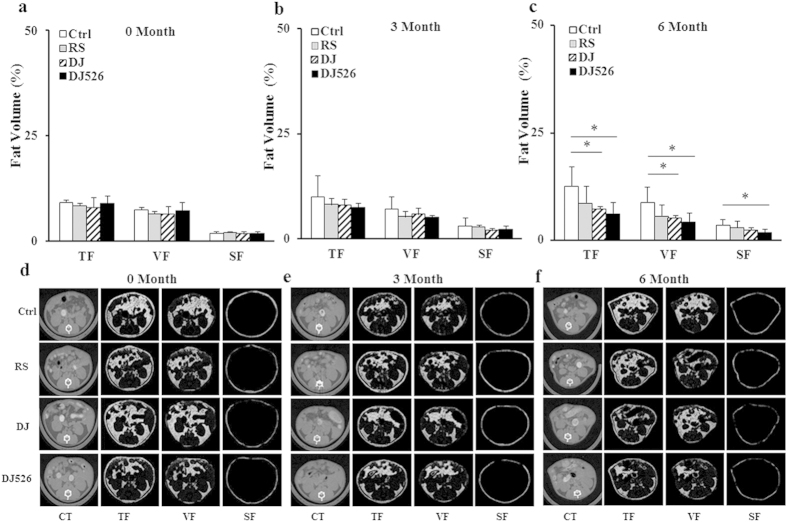
The effects of the resveratrol-enriched rice DJ526 on changes in abdominal fat content with age progression. The control (Ctrl) mice fed a NFD in which the carbohydrate source was corn starch and sucrose; The resveratrol (RS) mice fed a NFD in which the carbohydrate source was corn starch and sucrose except containing resveratrol; Dongjin (DJ) mice fed a NFD in which the corn starch and sucrose were replaced with Dongjin rice; DJ526, mice fed a NFD in which the corn starch and sucrose were replaced with the resveratrol enriched rice DJ526. The resveratrol concentration in DJ526 and RS groups was 0.69 μg/g each, and not detected in Ctrl and DJ groups. (**a–c**) Comparative analysis of the mice abdominal fat volume measured by *in vivo* micro-CT image analysis at 0 month (**a**), 3 month (**b**) and 6 month (**c**). (**d–f**) The representative micro-CT image used for abdominal fat analysis at 0 month (**d**), 3 month (**e**) and 6 month (**f**). The values represent the mean ± s.d. (n = 20). An unpaired Student’s t-test was used for the statistical analysis; *p < 0.05. TF, total fat; VF, visceral fat; SF, subcutaneous fat.

It has been well noted that an increase in body fat accumulation correlates with age progression^16^,^17^,^18^. Considering the fact that body weight generally reflects body fat, it would be reasonable to investigate whether inhibition of body weight gaining in Dongjin rice and the resveratrol-enriched rice DJ526 during age progression (Fig. 1) would result from inhibition of body fat accumulation. Total body fat, including its composition in each mouse group, was measured by a morphometric method using micro-CT. As shown in Fig. 2, both Dongjin rice and the resveratrol-enriched rice DJ526 efficiently inhibits accumulation of total body fat, visceral fat, and subcutaneous fat. At 6 months, the volumes of total fat, visceral fat, and subcutaneous fat in the DJ526 group were 6.27 ± 2.5%, 4.41 ± 1.9% and 1.83 ± 0.7%, respectively, which were significantly lower than the fat volumes of the control (12.6 ± 4.6%, 8.9 ± 3.6%, and 3.6 ± 1.3%, respectively) and the RS group (8.7 ± 3.9%, 5.7 ± 2.5%, and 3 ± 1.4%), and slightly lower than the DJ group (7.3 ± 0.6%, 5.1 ± 0.7%, and 2.3 ± 0.6%). Although the group difference between Dongjin rice and the resveratrol-enriched rice DJ526 was not statistically significant, the mice fed with the resveratrol-enriched rice DJ526 showed slightly lower fat accumulation. Other than fat volume, the measurement of fat mass index on the experimental mice also showed that Dongjin rice and the resveratrol-enriched rice DJ526 inhibited accumulation of body fat (see Supplementary Fig. S1). Overall, Dongjin rice and the resveratrol-enriched rice DJ526 efficiently inhibited accumulation of body fat, an important indicator of aging and general health.

### Mice fed Dongjin rice or resveratrol-enriched rice DJ526 maintained a healthy blood profile

**Figure 3:**
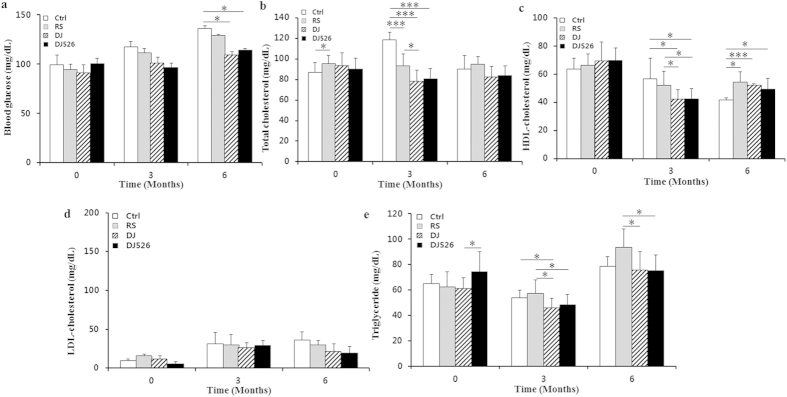
The effects of the resveratrol-enriched rice DJ526 on changes in blood with age progression. The control (Ctrl) mice fed a NFD in which the carbohydrate source was corn starch and sucrose; The resveratrol (RS) mice fed a NFD in which the carbohydrate source was corn starch and sucrose except containing resveratrol; Dongjin (DJ) mice fed a NFD in which the corn starch and sucrose were replaced with Dongjin rice; DJ526, mice fed a NFD in which the corn starch and sucrose were replaced with the resveratrol enriched rice DJ526. The resveratrol concentration in DJ526 and RS groups was 0.69 μg/g each, and not detected in Ctrl and DJ groups. Changes in Blood glucose levels (**a**), Total cholesterol levels (**b**), HDL-cholesterol levels (**c**), LDL-cholesterol levels (**d**) and Triglyceride levels (**e**) of mice during the 6 month experimental period. The values represent the mean ± s.d. (n = 20). Values in the figure with a superscripted letter indicate statistical significance as analyzed by an unpaired Student’s t-test; *p < 0.05, ***p < 0.001.

In mammals, in addition to body fat accumulation, the glucose and lipid levels in blood tend to increase during the course of aging^19^,^20^,^21^,^22^,^23^. Because Dongjin rice and the resveratrol-enriched rice DJ526 obviously inhibited body fat accumulation (Fig. 2 and see Supplementary Fig. S1), we investigated the effect of Dongjin rice and the resveratrol-enriched rice DJ526 on the blood profile. As shown in Fig. 3, Dongjin rice and the resveratrol-enriched rice DJ526 was effective in maintaining a healthy blood profile. The initial blood glucose level remained consistent during the experimental period at 3 months and 6 months in both of the DJ and DJ526 group. In contrast, the blood glucose level steadily increased during the 6-month experimental period from 99.4 ± 10.1 mg/dL to 136.4 ± 2.7 mg/dL in controls. Compared to the control, the overall blood profile was most well maintained in the DJ526 group and intermediately well maintained in the DJ groups although statistically not significant. Considering the fact that this experiment was conducted under normal diet conditions, not high calorie diet conditions, and the degree of difference in the blood profiles of the experimental groups was quite significant. While the blood glucose and lipid profile in the DJ and DJ526 group were lower than in the control, HDL cholesterol, the good kind of cholesterol, in the DJ and DJ526 group was higher than in the control. Overall, the blood profile analyses indicated that Dongjin rice and the resveratrol-enriched rice were effective in maintaining a healthy blood profile.

### The resveratrol-enriched rice DJ526 boosts motor coordination and physical strength

**Figure 4:**
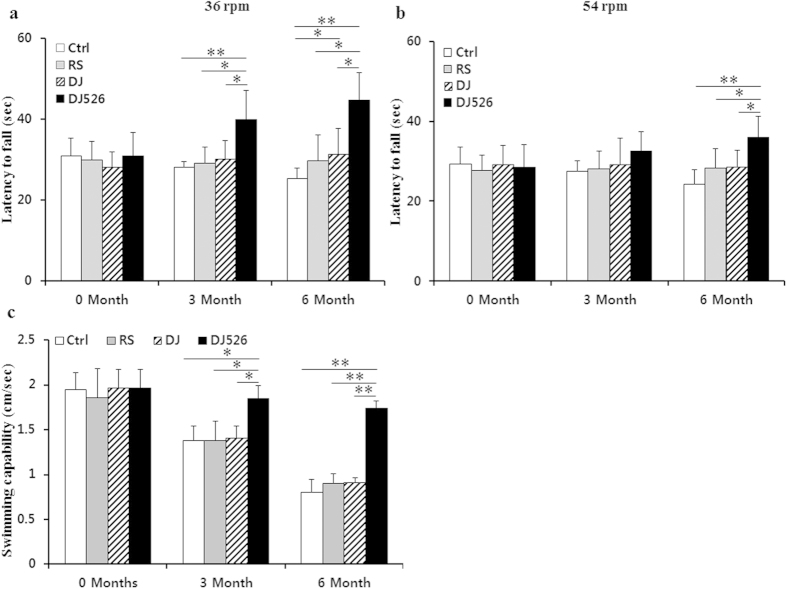
The effects of the resveratrol-enriched rice DJ526 on motor coordination and physical strength with age-progression. (**a,b**) The control (Ctrl) mice fed a NFD in which the carbohydrate source was corn starch and sucrose; The resveratrol (RS) mice fed a NFD in which the carbohydrate source was corn starch and sucrose except containing resveratrol; Dongjin (DJ) mice fed a NFD in which the corn starch and sucrose were replaced with Dongjin rice; DJ526, mice fed a NFD in which the corn starch and sucrose were replaced with the resveratrol enriched rice DJ526. The resveratrol concentration in DJ526 and RS groups was 0.69 μg/g each, and not detected in Ctrl and DJ groups. Performance of mice on the rotarod test at 36 and 54 rpm. (**c**) Performance of mice in the swimming test. Both tests were performed at 0, 3 and 6 month intervals. The values represent the mean ± s.d. (n = 20). Values in the figure with a superscripted letter indicate statistical significance as analyzed by an unpaired Student’s t-test; *p < 0.05, **p < 0.01.

It is well noted that paucities in motor coordination and physical strength are the phenotypic symptoms of old age^24^,^25^,^26^. Because the resveratrol-enriched rice DJ526 inhibited body fat accumulation and maintained a healthy blood profile during the aging process, most likely by ameliorating age-related deterioration, we investigated whether the resveratrol-enriched rice DJ526 inhibits paucities in motor coordination and physical strength during the aging progress using the fixed speed rotarod test and swimming test. The fixed speed rotarod test at both speeds of 36 rpm and 54 rpm showed that the resveratrol-enriched rice DJ526 significantly inhibits paucities in neuromuscular coordination compared to the control (Fig. 4a,b). At 6 months, the mice of the DJ526 group balanced themselves for 79% and 50% longer time at speeds of 36 rpm and 54 rpm, in comparison with the control group. Additionally, the swimming test showed that the resveratrol-enriched rice DJ526 delayed paucity of physical strength so that the mice of DJ526 group swam 2.2 times faster than the control (Fig. 4c). Overall, both experiments clearly indicate that the mice fed with resveratrol-enriched rice DJ526 exhibited significant increments in motor coordination and physical strength in older age, in contrast to the control. Dongjin rice was slightly effective in delaying paucities in neuromuscular coordination and physical strength while resveratrol and Dongjin rice were slightly effective. It is very interesting to note that the resveratrol-enriched rice DJ526 is effective in maintaining healthy blood profile, body weight, neuromuscular coordination, and physical strength while the efficacy of Dongjin rice is limited to maintaining healthy levels of body weight and blood profile.

### Resveratrol-enriched rice DJ526 prevents aging of tissues

**Figure 5:**
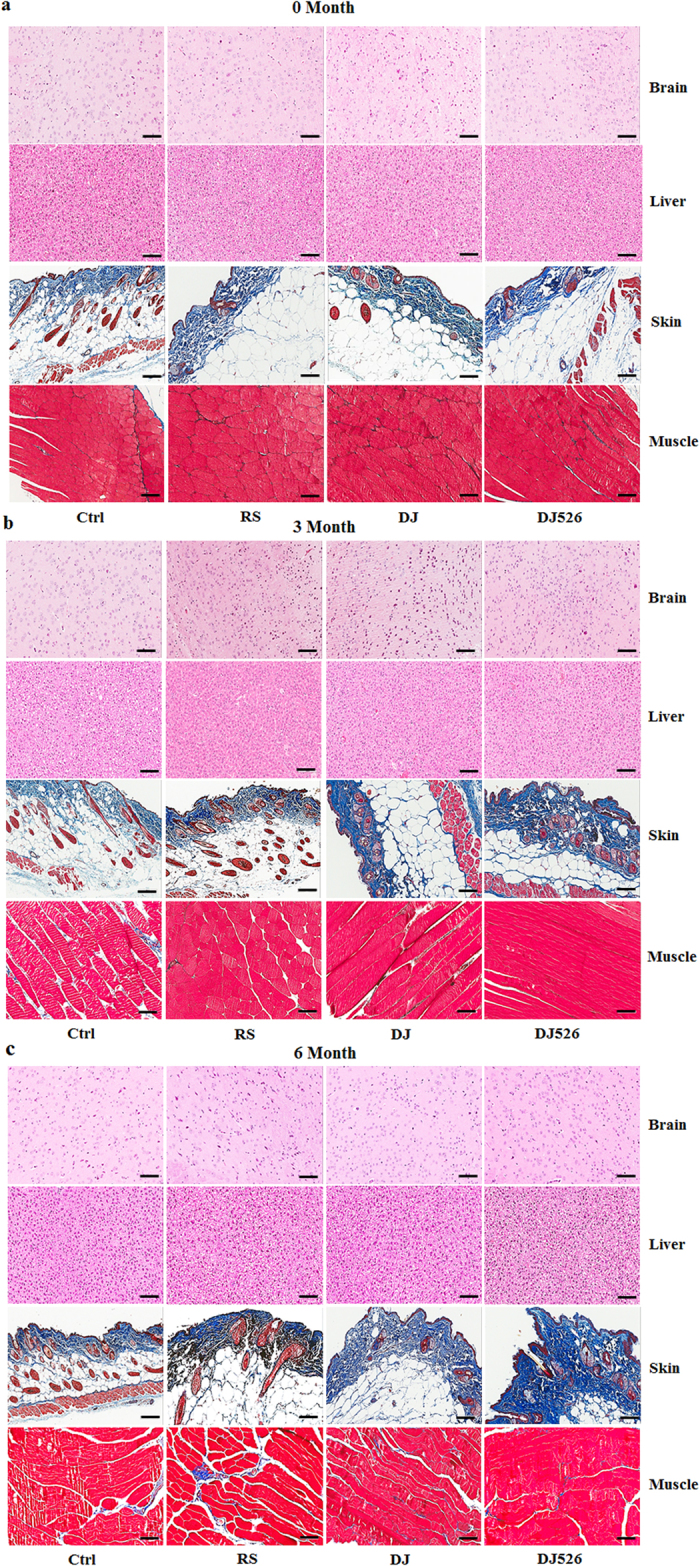
The effects of the resveratrol-enriched rice DJ526 in organs with age-progression. The control (Ctrl) mice fed a NFD in which the carbohydrate source was corn starch and sucrose; The resveratrol (RS) mice fed a NFD in which the carbohydrate source was corn starch and sucrose except containing resveratrol; Dongjin (DJ) mice fed a NFD in which the corn starch and sucrose were replaced with Dongjin rice; DJ526, mice fed a NFD in which the corn starch and sucrose were replaced with the resveratrol enriched rice DJ526. The resveratrol concentration in DJ526 and RS groups was 0.69 μg/g each, and not detected in Ctrl and DJ groups. Brain and liver histology images with H&E stain. Skin and muscle histology images with MT stain. Representative images of each organ from four experimental groups at 0 month (**a**), 3 month (**b**) and 6 month (**c**). Scale bars, 100 μm in all the images.

**Figure 6:**
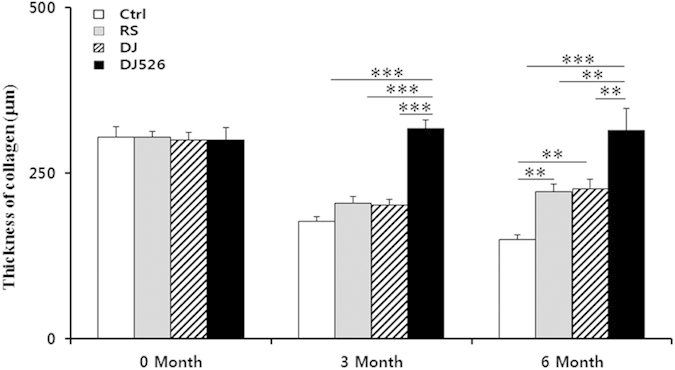
The effects of the resveratrol-enriched rice DJ526 on changes in the medial thickness of collagen at 0 month, 3 month and 6 months. The control (Ctrl) mice fed a NFD in which the carbohydrate source was corn starch and sucrose; The resveratrol (RS) mice fed a NFD in which the carbohydrate source was corn starch and sucrose except containing resveratrol; Dongjin (DJ) mice fed a NFD in which the corn starch and sucrose were replaced with Dongjin rice; DJ526, mice fed a NFD in which the corn starch and sucrose were replaced with the resveratrol enriched rice DJ526. The resveratrol concentration in DJ526 and RS groups was 0.69 μg/g each, and not detected in Ctrl and DJ groups. The values represent the mean ± s.d. (n = 6). Values in the figure with a superscripted letter indicate statistical significance as analyzed by an unpaired Student’s t-test; **p < 0.01, ***p < 0.001.

Because all of the above experiments indicate that the resveratrol-enriched rice DJ526 prevents age-related deterioration, we observed the effect of long-term feeding of the resveratrol-enriched rice DJ526 at the tissue level. As shown in Fig. 5, Hematoxylin and Eosin (H&E) staining examinations on brain and liver did not show any significant difference among different experimental groups, although the brain and liver tissues of the DJ526 mice seemed to be slightly healthier than in other mice. However, Masson trichrome (MT) staining examination on muscle showed that the muscle of the DJ526 mice was much more compact than other mouse groups. MT staining examination on skin also showed that the collagen layer of the DJ526 mice was much thicker than in other mouse groups. These MT staining results indicate that the mice fed with resveratrol-enriched rice DJ526 were much healthier than the control, meaning that the resveratrol-enriched rice DJ526 inhibited aging of muscle and skin. Compared to the control, the skin tissue sections stained with MT showed a thick and healthy collagen layer in the epithelium and subepithelium of the DJ526 mice (Fig. 5). Because previous studies showed that the quantity of collagen in skin is an important indicator of aging^27^,^28^,^29^,^30^,^31^, the medial thickness of the collagen in skin was quantitated using the Image-J system. The medial thickness of collagen in skin after the 3-month experimental period was 327.8 ± 13.4 μm in the DJ526 group, 203.3 ± 7.5 μm in the DJ group, 204.4 ± 10.2 μm in the RS group, and 177.8 ± 7.0 μm in the control group (Fig. 6). The collagen layer of DJ526 group was 1.8−2.0-fold higher than in the control, which is statistically significant to conclude that the resveratrol-enriched rice DJ526 prevents skin aging. As shown in Fig. 6, the anti-aging effect of the resveratrol-enriched rice DJ526 was maintained until 6 months, in which the collagen layer of DJ526 group was 1.8−2.5-fold higher than in the control. Again, it should be noted that only the resveratrol-enriched rice DJ526, but not Dongjin rice, is very significantly effective in anti-aging.

### The gene expression pattern of mice fed the resveratrol-enriched rice DJ526 was very different from mice fed either resveratrol or Dongjin rice alone, respectively

**Figure 7:**
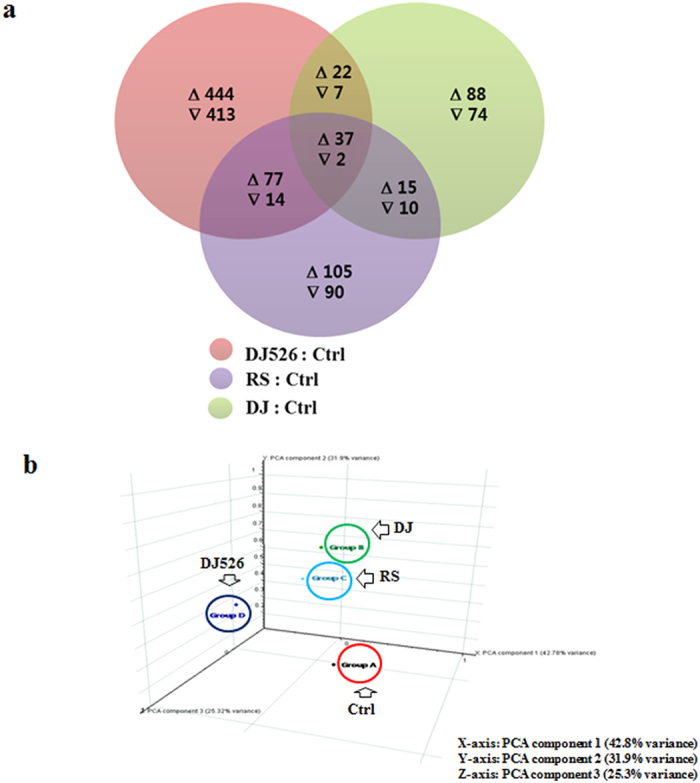
The gene expression patterns of the mice on resveratrol (RS), Dongjin rice (DJ), resveratrol-enriched rice DJ526 (DJ526), or control (Ctrl) diet. The control (Ctrl) mice fed a NFD in which the carbohydrate source was corn starch and sucrose; The resveratrol (RS) mice fed a NFD in which the carbohydrate source was corn starch and sucrose except containing resveratrol; Dongjin (DJ) mice fed a NFD in which the corn starch and sucrose were replaced with Dongjin rice; DJ526, mice fed a NFD in which the corn starch and sucrose were replaced with the resveratrol enriched rice DJ526. The resveratrol concentration in DJ526 and RS groups was 0.69 μg/g each, and not detected in Ctrl and DJ groups. (**a**) Overlaps of differential expression signatures at the end of 6 month feeding experiments. A total of 1016 probe sets (<2.6%) were induced by DJ526/Ctrl, while 255 probe sets (<0.7%) were induced by DJ/Ctrl and 350 probe sets (<0.9%) were induced by RS/Ctrl. The Venn diagram shows the overlap between these groups, where up and down triangles indicate the number of probe sets up and down-regulated, respectively. Genes were filtered using a cutoff Z-ratio ± 1.5 and p-value of <0.05. (**b**) Principal component analysis of the microarray data at the end of the 6 month feeding experiments. The first principal component (PC1) is dominant with 42.8% variability, and shows that DJ526 is different from DJ, RS, or Ctrl, respectively.

As shown in Figs 4−6 and previous works^10^,^11^, a synergistic combination of the innate properties of Dongjin rice and the transgenic properties of the resveratrol-enriched rice DJ526 showed unexpectedly higher beneficial health effects than was expected. A possible explanation of these superior health effects of the resveratrol-enriched rice DJ526 is that the innate components of Dongjin rice and the transgenic properties of resveratrol synergistically affect biochemical or signaling pathways that lead to superior health benefits overall. To confirm this hypothesis, we performed whole-genome microarrays and pathway analyses on the liver samples of mice fed with the resveratrol-enriched rice DJ526, Dongjin rice, resveratrol alone, and control. The Z ratios for the pairs of groups were calculated as described previously^32^ and a subset of expression changes was confirmed by quantitative real time PCR (see Supplementary Fig. S3). As shown in Supplementary Fig. S3, the levels of gene expression measured by either quantitative real time PCR or the whole-genome microarray method were basically identical. The overall gene expression pattern of the DJ526 group was much more significantly changed than group DJ or RS. Expression patterns for 1016 out of 39,429 (<2.6%) individual genes changed significantly relative to the control in the DJ526 group (Individual genes with absolute value for the Z-ratio > 1.5 and p-value < 0.05 were considered significantly changed), while 255 (<0.7%) and 350 (<0.9%) out of 39,429 individual genes changed significantly relative to the control in the DJ and RS groups respectively (Fig. 7a). It is worth noting that the global transcriptional profile in the DJ526 group was much more significantly changed than the RS or DJ group. The ten most up-regulated and down-regulated genes in each of the RS, DJ, and DJ526 group are listed in Supplementary Table S2. As expected from the results of physiological analysis (Figs 1, 2, 3, 4, 5, 6), the global transcriptional profiles of each of the three groups were different from each other (Fig. 7a and see Supplementary Table S2).

To test the hypothesis that the physiological effects of DJ526 are different from either DJ or RS, we performed principal component analysis (PCA) on microarray data from each sample. Each principal component (PC) roughly represents a set of correlated changes in gene expression and is independent of every other PC. PCs are ranked based on the contribution, and each PC makes the total variability between samples^33^,^34^. PCA yielded values of −0.85 (DJ526), −0.2 (DJ), −0.38 (RS), and +0.18 (Ctrl), with 42.8% of variability assigned to the first PC. The PCA analyses showed that the effects on gene expression profiles from feeding DJ526 were different from the gene expression profiles from feeding either DJ or RS, although the physiological effects of RS and DJ were similar. Interestingly, the PCA analyses showed that changes in the gene expression profiles from feeding RS or DJ were quite similar to each other, as expected from global physiological effects. However, changes in gene expression profiles from DJ526 feeding were different from gene expression profiles of either the DJ group or the RS group.

**Figure 8:**
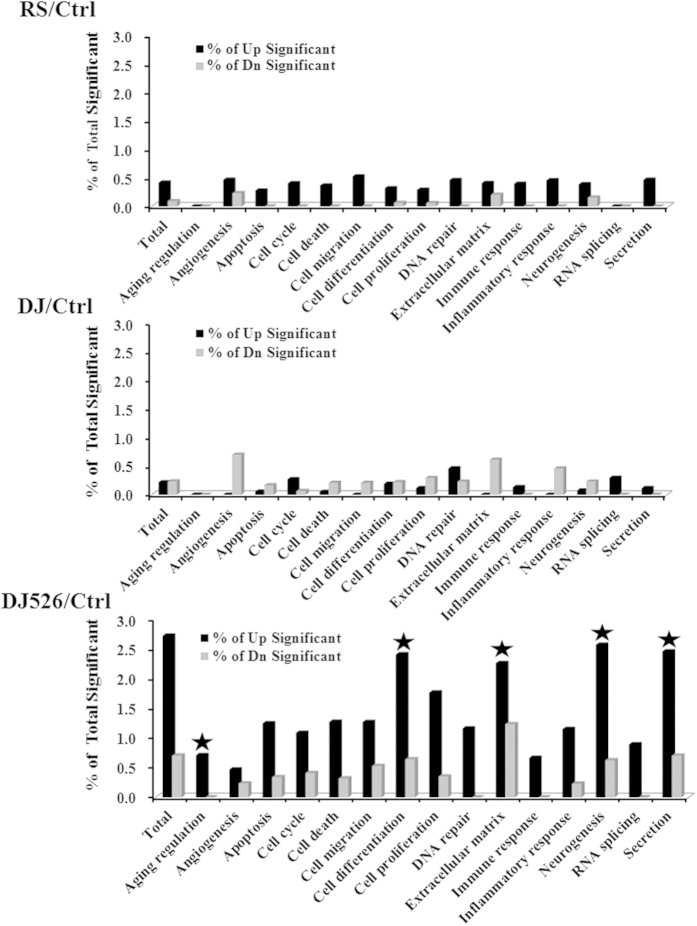
The gene ontology analyses of the microarray data from the mice on resveratrol (RS), Dongjin rice (DJ), resveratrol-enriched rice DJ526 (DJ526), or control (Ctrl) diet. The control (Ctrl) mice fed a NFD in which the carbohydrate source was corn starch and sucrose; The resveratrol (RS) mice fed a NFD in which the carbohydrate source was corn starch and sucrose except containing resveratrol; Dongjin (DJ) mice fed a NFD in which the corn starch and sucrose were replaced with Dongjin rice; DJ526, mice fed a NFD in which the corn starch and sucrose were replaced with the resveratrol enriched rice DJ526. The resveratrol concentration in DJ526 and RS groups was 0.69 μg/g each, and not detected in Ctrl and DJ groups. Each panel represents the gene ontology data which were analyzed from the microarray data at the end of 6 month feeding experiments. The gene ontology analyses were performed from the microarray data filtered using cutoff a fold-change of >1.5 and p value of <0.05. The most significantly altered gene expression patterns by DJ526 treatment are indicated in symbol (★).

We next performed gene set enrichment of analysis (GSEA) to investigate differences among experimental groups. A gene set of 1045 pathways was obtained from the Broad Institute Database and analyzed as described previously^35^,^36^. Pathways that were significantly altered by treatment of DJ526, DJ, or RS are represented in Supplementary Fig. S2. The effects of DJ and RS treatments had 37.9% correlation (by direction of change), while DJ and DJ526 had 27.3% correlation, and DJ526 and RS had a correlation of 50.1% (see Supplementary Fig. S2). The GSEA data again indicated that the effects of the DJ526 treatments on global gene expression profiles were different from those of the DJ or RS treatments, indicating that DJ526 is not simply an addition of DJ and RS. The top 12 most highly elevated or 12 down-regulated pathways in each group were listed in Supplementary Table S3. Notably, the change in gene expression profiles in the resveratrol-fed group were similar to previously reported resveratrol treatment experiments^37^,^38^,^39^,^40^,^41^, including the most significantly up-regulated pathways such as immunological and inflammatory reactions and the most down-regulated pathways such as apoptosis, fatty acid synthesis, and ubiquitin ligase by resveratrol treatment (see Supplementary Table S3). However, the DJ526-fed group showed different gene expression profiles from either resveratrol or DJ treatment. To investigate the detailed physiological effects by DJ, RS, or DJ526 treatment, we selected significantly changed genes (absolute value of fold-change of >1.5 and p-value of <0.05), and these genes were grouped based on gene ontology using the Gene Ontology Consortium database (www.geneontology.org/Ref.MouseDB). As shown in Fig. 8, the gene expression pattern by DJ526 treatment differed from either DJ or RS treatment. It should be noted that the genes related to aging regulation, cell differentiation, extracellular matrix, neurogenesis, or secretion were altered most significantly in the DJ526 treatment.

## Discussion

Resveratrol is a non-flavonoid polyphenol-type stilbene compound produced naturally by several plants in response to environmental stresses^42^. Although resveratrol has been reported to show various beneficial health effects, its main function is to delay age-related diseases^43^,^44^ by inhibiting cAMP-dependent phosphodiesterases to trigger a cascade of events including AMPK (AMP-activated protein kinase), SIRT1 (Silent mating type information regulation 2, S. cerevisiae, homolog 1), and PGC-1α (Peroxisome proliferator-activated receptor-gamma coactivator 1α)^45^. Despite the fact that resveratrol extends the lifespan of yeast, worms, fruit flies, and fish, the effect of resveratrol on lifespan in rodent models has not been reproduced^37^,^38^,^39^,^40^,^41^,^44^,^46^,^47^,^48^,^49^,^50^,^51^,^52^. However, there is some beneficial evidence of resveratrol in mice from mimicking the ability of dietary restriction to delay the physiological deterioration associated with aging^37^,^38^,^39^,^40^,^41^.

It has been shown that long-term resveratrol treatment of mice mimics transcriptional changes induced by dietary restriction^37^,^38^,^39^,^40^,^41^. However, resveratrol did not mimic all of the salutary effects of dietary restriction and did not increase longevity^37^. The gene expression profiling analyses in this work showed that the gene expression pattern of the DJ526 treated group was completely different from those of either DJ or RS. In particular, compared to the DJ or RS groups, aging regulation and extracellular matrix pathways in the DJ526 group were very significantly up-regulated. Up-regulation of extracellular matrix can explain the healthy thickness of collagen in the cutaneous layer in the DJ526 treated group (Fig. 6). It has been well known that a thick collagen layer in the body indicates youth in mammals, and the collagen layer becomes thinner as it ages^27^,^28^,^29^,^30^,^31^. Therefore, the healthy thickness of collagen as well as up-regulation of anti-aging related pathways in the DJ526 treated group can explain why the DJ526-treated mice were vastly superior in physical strength than the RS or DJ treated group.

The biochemical pathways of lower animals are generally well-conserved in higher animals including humans, and thus, the effects of small molecules are also frequently conserved^53^,^54^. Considering the general tendency of evolutionary conservation of biochemical pathways, it is expected that resveratrol expands the lifespan of mice as in the case of lower animals. However, the efficacy of resveratrol on lifespan in lower animals was diminished in mice^37^. The beneficial effects of resveratrol in delaying the physiological deterioration associated with aging were limited to partial mimicry of dietary restriction without extending lifespan^37^. As shown in Figs 1−6, this work agrees with previous studies that the beneficial effects of resveratrol are limited. However, the DJ526 was much more beneficial and anti-aging than RS. Considering that completely different gene expression profiling leads to pathways related to anti-aging, it is reasonable to speculate that DJ526 would increase the lifespan of mammals, which would not be possible with resveratrol alone.

Parental plant of the resveratrol-enriched rice DJ-526, *Oryza sativa* japonica variety Dongjin rice developed by the Rural Development Administration of Korea, has characteristics of having grains rich in polyphenols with anti-obesity activity compared to other rice variety^11^. We believe that the different biochemical pathways triggered independently by resverarol and polyphenols in the resveratrol-enriched rice DJ-526 (Figs 7 and 8) might synergistically work together to bestow unexpectedly high health-beneficial effects. Further detailed researches on the biochemical pathways are needed to definitely conclude the hypothesis.

Functional foods, providing additional health benefits in addition to nutrition, have great potential to affect healthcare of mankind by preventing or treating diseases using foods. Our work shows for the first time that consideration of a synergistic effect in creating a GM crop could lead to the generation of unexpectedly beneficial health effects that can be used to prevent and/or treat various age-related diseases.

## Methods

### Animal experiments

C57BL/6 female mice were purchased from Joongang Experimental Animal Co. (Seoul, Korea) at six weeks of age. The mice were housed 10 animals per cage, with food (10% kcal as fat; D12450B; Research Diets Inc., New Brunswick, NJ, USA) and water available ad libitum unless otherwise stated. They were maintained under a 12 h light/12 h dark cycle at a temperature of 22 ± 1 °C and humidity of 40–50%. After 2 weeks of acclimation, a total of 80 mice were randomly divided into the following groups: Normal Formula Diet (Ctrl), NFD supplemented with resveratrol (RS), NFD in which the corn starch and sucrose were replaced with Dongjin rice (DJ), NFD in which the corn starch and sucrose were replaced with resveratrol-enriched rice DJ526 (DJ526). The composition of the diets is given in Supplementary Table S1. The resveratrol concentration was quantified in all formula diets by HPLC (ACQUITY UPLC, Waters, MA, USA) as described previously^10^,^11^. The food consumption of each mouse group was monitored on a daily basis.

All animal care and use were performed strictly in accordance with the ethical guidelines by the Ethics Committee of Chonbuk National University Laboratory Animal Center, and the animal study protocol was approved by the institution.

### *In vivo* efficacy assay

The blood glucose and lipid levels were measured at 0, 3 and 6 months during treatment. The food consumption of each mouse group was regularly monitored. Blood samples were drawn from the tail after 5 h of fasting, and blood-glucose measurements were taken using an Accu-check Glucometer (Roche, Basel, Switzerland). The serum was separated by centrifuging at 13,000 rpm for 10 min and immediately stored at 22 °C until assayed. The triglycerides, total cholesterol and HDL-cholesterol levels in the serum were quantitatively determined using enzymatic colorimetric methods (Asan Pharmaceutical, Seoul, Korea).The LDL-cholesterol levels were calculated using the Friedewald equation [(LDL) = (T-CHO) − (HDL) −(TG)/5]. The body weights were measured at 0, 3 and 6 months after treatment. For fat analysis, the total body fat was determined by high-resolution *in vivo* micro-CT (Skyscan, Konitch, Belgium) with a high resolution CCD/phosphor screen detector. Before CT, the mice were anesthetized with zoletil and rumpun (4:1) and placed on a radio-transparent mouse bed in a supine position with the caudal end closest to the micro-CT. The hind legs were extended and held in place with clear tape to ensure the correct anatomical position. Micro-CT images of the abdomen were captured at the level of the L1–L5 inter-vertebral disks, and the total fat, visceral fat and subcutaneous fat areas were analyzed using CTan Ver.1.10, Skyscan software (Skyscan, Konitch, Belgium). Analysis of the fat mass index was calculated using the fat mass/total body weight (g/g) of mice.

### Behavioral test

The mice of four groups at 0, 3 and 6 months age were trained and then tested on a battery of behavioral tests that were comprised of swim tank (swimming, motivation and coordination) and rotarod (coordination, balance and neuro-muscular strength) tests^55^,^56^. **Swim tank test**: To monitor swimming ability and efficiency, the mice were trained to swim from one end of a water-filled glass tank to a visible escape platform at the opposite end. The glass tank (40 × 25 × 16 cm^3^) was filled to a depth of 20 cm with water maintained at a temperature of 23 °C. To monitor their swimming ability, the mice of four groups were placed at one end of the water-filled glass tank with a visible escape platform at the opposite end to a maximum of 120 sec. The mice that were unable to swim were guided by the hand to the escape platform, placed on the platform and allowed to remain there for 15 sec, before being removed and returned to the home cage. **Rotarod test:** The rotarod apparatus (ROTA ROD, Haryana, India) was used to assess motor coordination, strength and balance. The apparatus consisted of a base platform and a rotating rod with a diameter of 3 cm and a non-slippery surface. The rod was placed at a height of 15 cm from the base. The rod, 30 cm in length, was divided into four equal sections by three fiber plates. Thus, up to four mice were tested simultaneously on the apparatus, with the same rod rotating speed. The mice of four groups received four trials per day, at 36 rpm for three consecutive days. On the fourth day, mice underwent testing. During testing, each animal received three trials at two increased speeds (36 and 54 rpm). The mean latency to fall off the rotarod was recorded and used in the subsequent analysis.

### Histology

Brain, liver, muscle and skin tissues were isolated from postmortem mice at 0, 3 and 6 months for histological examination from all experimental groups. Immediately after isolation, all tissues were fixed at 10% neutral-buffered formalin, embedded in paraffin and sectioned (5 μm). Paraffin was removed from the tissue section by hot water and placed on a glass microscopic slide and the slides were air dried and baked overnight at 65 °C. The brain and liver tissue sections were stained with Hematoxyline and Eosin (H&E), and the skin and muscle tissue section were stained with Masson trichrome (MT) with standard staining procedure. The stained tissue images were observed in Apero ScanScope FL (Leica Biosystems, Nussloch, Germany), the medial thickness was measured using the Image-J system (NIH, Maryland, USA), and the mean of the four measurements was taken as the thickness of the media.

### Microarray -Z scores, GSEA and PCA

Three mice per group at 6 months of age were sacrificed and livers were flash frozen. Approximately 100 mg of each liver was used for RNA extraction by RNeasy Mini kit according to the manufacturer’s protocol (Qiagen, Limburg, Netherlands). RNA was extracted, amplified, labeled, and hybridized to Agilent Gene Expression hybridization kit (Agilent Technologies, CA, USA) according to the manufacturer’s instructions. Z normalization and tests for significant changes were done as previously described^32^. Raw data were subjected to Z normalization to ensure compatibility using the formula:


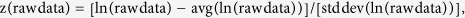


where ln is natural logarithm, avg is the average over all genes of an array, std dev is the standard deviation over all genes of an array. The Z ratio (between treatment A and B) is given by z(A) − z(B)/std dev. Individual genes with Z ratio > 1.5, *P* value < 0.05, and avg intensity > 0 were considered significantly changed.

For gene set enrichment of analysis (GSEA), a set of 1045 pathway was obtained from http://www.broadinstitute.org/gsea/downloads.jsp (C2.all.v5.0.symbols.gmt). Gene set enrichment was compared in each of the four groups, Ctrl, DJ, RS and DJ526. Gene set enrichment analysis (GSEA) was performed using GSEA v2.2.0^35^,^36^. Principal component analysis (PCA) was performed on the replicate average for the four groups. We examined the dominant first principal component (PCA1) in order to compare DJ526, Ctrl, DJ and RS-fed mice, respectively. Principal components analysis (PCA) was performed using GeneSpringGX 7.3 (Agilent Technology, CA, USA). Gene classification was based on Database for Annotation, Visualization and Integrated Discovery (DAVID) web-accessible program (http://david.abcc.ncifcrf.gov/). Heatmap was analyzed using MultiExperiment Viewer (The Institute for Genomic Research, http://www.tm4.org/mev/) version 4.8.1.

### Quantitative real-time PCR

Total RNAs from the liver samples were isolated with RNeasy Mini kit according to the manufacturer’s protocol (Qiagen, Limburg, Netherlands) and were reverse transcribed using Superscript^TM^ II RT (Invitrogen, Ca, USA). Real–time RT-PCR was used to analyze mRNA expression (n = 3 for each group) using the StepOnePlus^TM^ (Applied Biosystems, CA, USA). Quantification was performed using the ∆∆CT method. The housekeeping gene, Glyceraldehyde-3-Phosphate Dehydrogenase, was used for internal normalization. Primer sequences are listed in Supplementary Table S4.

### Statistical analysis

All data were expressed as the mean ± s.d., as indicated. The statistical comparisons were analyzed using an unpaired Student’s t-test. All differences were considered statistically significant if p < 0.05. Statistical significance is shown as *p < 0.05, **p < 0.01 and ***p < 0.001.

## Additional Information

**How to cite this article**: Chung, H.-J. *et al*. The resveratrol-enriched rice DJ526 boosts motor coordination and physical strength. *Sci. Rep.* **6**, 23958; doi: 10.1038/srep23958 (2016).

## Supplementary Material

Supplementary Information
