# Supplementary material for: The resveratrol-enriched rice DJ526 boosts motor coordination and physical strength
Source: Sci Rep. 2016 Apr 5;6:23958. doi: 10.1038/srep23958 (PMC4820726; doi:10.1038/srep23958)
Supplement: Supplementary Information [file srep23958-s1.pdf]

SUPPLEMENTARY INFORMATION

The resveratrol-enriched rice DJ526 boosts motor coordination and physical strength

Hea-Jong Chung[1, †], So-Hyeon Baek[2, †], Satya Priya Sharma[1], Hyeon-Jin Kim[3], Seong-Tshool Hong[1, *]

Supplementary Tables 1, 2, 3, 4

Supplementary Figure 1, 2, 3 and legends

Supplementary Tables

Supplementary Table S1: The compositional variation of the Formula Diet fed to the experimental mice

Ingredient	Ctrl	RS	DJ	DJ526
Casein	200	200	200	200
L-Cysteine	3	3	3	3
**Corn Starch**	**397.486**	**397.486**	**0**	**0**
**Sucrose**	**100**	**100**	**0**	**0**
Maltodextrin	132	132	132	132
Soybean Oil	70	70	70	70
Cellulose	50	50	50	50
Mineral Mix, AIN-93G-MX (94046)	35	35	35	35
Vitamin Mix, AIN-93-VX (94047)	10	10	10	10
Choline Bitartrate	2.5	2.5	2.5	2.5
TBHQ, Antioxidant	0.014	0.014	0.014	0.014
**Resveratrol**	**0**	**0.00098**	**0**	**0**
**Dongjin Rice**	**0**	**0**	**497.486**	**0**
**Dongjin-526 Rice**	**0**	**0**	**0**	**497.486**
Total (g)	1000	1000	1000	1000

All diet formulations were based on the diet formula of nonfat normal diet (TD.94045 AIN-93G) of Raonbio (Seoul, Korea), which is the control diet in this experiment. Deviations from the control are indicated in bold. The resveratrol concentration in all of the formula diets was quantified by HPLC (ACQUITY UPLC, Waters, MA, USA). The resveratrol quantities were 0.69 µg/g in resveratrol-enriched DJ526 diet (DJ526) and resveratrol diet (RS) each. However, resveratrol was not detected in control diet (Ctrl) and dongjin rice diet (DJ).

Supplementary Table S2: Differentially expressed genes by effect of resveratrol, Dongjin rice, and the resveratrol-enriched rice DJ526 (DJ526)

Gene	Z ratios RS/Ctrl
Sult2a4	11.07
Sult2a1	9.13
Sult2a3	8.36
Gm6168	7.30
Sult2a6	6.77
Ap3b2	5.63
2310038E17Rik	5.33
Gm11435	5.16
Cd70	5.12
Olfr1106	5.07

Gene	Z ratios RS/Ctrl
Pde5a	-11.02
Dirc2	-6.80
Serpina4-ps1	-6.73
Emilin3	-6.08
Crygf	-5.55
Dnahc7a	-5.53
Gypa	-5.40
Bves	-4.71
Olfr681	-4.47
Pbx1	-3.93

Gene	Z ratios DJ/Ctrl
Csf3	5.53
Sub1	5.36
Olfr467	5.03
Serpinb1c	4.95
Ccnb1ip1	4.93
4930578C19Rik	4.93
D330041H03Rik	4.35
Dctd	4.28
H2-M5	4.21
Olfr915	3.95

Gene	Z ratios DJ/Ctrl
Duoxa1	-5.88
Pnoc	-5.68
Olfr877	-4.49
EG668249	-4.48
Prdm14	-4.47
Shh	-4.22
Olfr1513	-4.03
2410012M07Rik	-4.00
Myh13	-3.71
Chn1	-3.61

Gene	Z ratios DJ526/Ctrl
Sult2a6	5.65
Ap3b2	5.13
Tnfaip6	5.08
Tas1r2	4.88
Arsi	4.77
Olfr119	4.62
Trim43b	4.57
H2-M5	4.39
Olfr569	4.37
Usp54	4.21

Gene	Z ratios DJ526/Ctrl
Gpr31c	-7.46
Spock1	-6.72
Olfr638	-6.29
Olfr453	-6.03
Fgf15	-5.97
Gm4050	-5.76
Dnahc1	-5.73
2810049E08Rik	-5.64
Gm14003	-5.42
Gm4794	-5.19

The most highly significant up-regulated and down-regulated genes in the livers of mice on resveratrol diet (RS), dongjin rice diet (DJ) and resveratrol-enriched DJ526 diet (DJ526) towards those on a control diet (Ctrl) groups are shown. Genes were filtered using a cutoff Z-ratio ± 1.5 and P-value of < 0.05. Genes meeting these criteria were ranked by Z-ratio.

Supplementary Table S3: The most highly significant up-regulated and down-regulated pathways in the livers of mice on resveratrol diet (RS), Dongjin rice diet (DJ) and the resveratrol-enriched DJ526 diet (DJ526) towards those on the control diet (Ctrl) groups

	**RS/Ctrl**
**Up**	gene_sets.gmt#BIOCARTA_NO2IL12_PATHWAY
	gene_sets.gmt#KEGG_INTESTINAL_IMMUNE_NETWORK_FOR_IGA_PRODUCTION
	gene_sets.gmt#REACTOME_KINESINS
	gene_sets.gmt#KEGG_TASTE_TRANSDUCTION
	gene_sets.gmt#PID_TCR_CALCIUM_PATHWAY
	gene_sets.gmt#PID_IL12_2PATHWAY
	gene_sets.gmt#PID_IL12_STAT4_PATHWAY
	gene_sets.gmt#BIOCARTA_INFLAM_PATHWAY
	gene_sets.gmt#PID_CD8_TCR_DOWNSTREAM_PATHWAY
	gene_sets.gmt#BIOCARTA_CTLA4_PATHWAY
	gene_sets.gmt#PID_AURORA_B_PATHWAY
	gene_sets.gmt#BIOCARTA_NKT_PATHWAY
**Down**	gene_sets.gmt#BIOCARTA_PROTEASOME_PATHWAY
	gene_sets.gmt#HALLMARK_MYC_TARGETS_V1
	gene_sets.gmt#REACTOME_DESTABILIZATION_OF_MRNA_BY_AUF1_HNRNP_D0
	gene_sets.gmt#KEGG_BIOSYNTHESIS_OF_UNSATURATED_FATTY_ACIDS
	gene_sets.gmt#REACTOME_REGULATION_OF_APOPTOSIS
	gene_sets.gmt#HALLMARK_MTORC1_SIGNALING
	gene_sets.gmt#REACTOME_VIF_MEDIATED_DEGRADATION_OF_APOBEC3G
	gene_sets.gmt#REACTOME_AUTODEGRADATION_OF_THE_E3_UBIQUITIN_LIGASE_COP1
	gene_sets.gmt#REACTOME_RORA_ACTIVATES_CIRCADIAN_EXPRESSION
	gene_sets.gmt#REACTOME_CROSS_PRESENTATION_OF_SOLUBLE_EXOGENOUS_ANTIGENS_ENDOSOMES
	gene_sets.gmt#PID_ECADHERIN_KERATINOCYTE_PATHWAY
	gene_sets.gmt#HALLMARK_CHOLESTEROL_HOMEOSTASIS

	**DJ/Ctrl**
**Up**	gene_sets.gmt#KEGG_PRIMARY_IMMUNODEFICIENCY
	gene_sets.gmt#REACTOME_DEGRADATION_OF_THE_EXTRACELLULAR_MATRIX
	gene_sets.gmt#PID_CD8_TCR_DOWNSTREAM_PATHWAY
	gene_sets.gmt#BIOCARTA_NO2IL12_PATHWAY
	gene_sets.gmt#ST_TUMOR_NECROSIS_FACTOR_PATHWAY
	gene_sets.gmt#PID_IL12_2PATHWAY
	gene_sets.gmt#REACTOME_BASE_EXCISION_REPAIR
	gene_sets.gmt#REACTOME_NEF_MEDIATES_DOWN_MODULATION_OF_CELL_SURFACE_RECEPTORS_BY_RECRUITING_THEM_TO_CLATHRIN_ADAPTERS
	gene_sets.gmt#KEGG_BASE_EXCISION_REPAIR
	gene_sets.gmt#PID_AURORA_B_PATHWAY
	gene_sets.gmt#BIOCARTA_TNFR2_PATHWAY
	gene_sets.gmt#BIOCARTA_HIVNEF_PATHWAY
**Down**	gene_sets.gmt#KEGG_MATURITY_ONSET_DIABETES_OF_THE_YOUNG
	gene_sets.gmt#REACTOME_SIGNALING_BY_NODAL
	gene_sets.gmt#REACTOME_REGULATION_OF_GENE_EXPRESSION_IN_BETA_CELLS
	gene_sets.gmt#PID_CONE_PATHWAY
	gene_sets.gmt#KEGG_GLYCOSPHINGOLIPID_BIOSYNTHESIS_LACTO_AND_NEOLACTO_SERIES
	gene_sets.gmt#PID_RHODOPSIN_PATHWAY
	gene_sets.gmt#BIOCARTA_P53HYPOXIA_PATHWAY
	gene_sets.gmt#PID_PI3K_PLC_TRK_PATHWAY
	gene_sets.gmt#BIOCARTA_SHH_PATHWAY
	gene_sets.gmt#REACTOME_ACTIVATED_AMPK_STIMULATES_FATTY_ACID_OXIDATION_IN_MUSCLE
	gene_sets.gmt#REACTOME_REGULATION_OF_BETA_CELL_DEVELOPMENT
	gene_sets.gmt#BIOCARTA_PGC1A_PATHWAY

	DJ526/Ctrl
**Up**	gene_sets.gmt#KEGG_TASTE_TRANSDUCTION
	gene_sets.gmt#KEGG_INTESTINAL_IMMUNE_NETWORK_FOR_IGA_PRODUCTION
	gene_sets.gmt#PID_CD8_TCR_DOWNSTREAM_PATHWAY
	gene_sets.gmt#PID_NFAT_TFPATHWAY
	gene_sets.gmt#HALLMARK_E2F_TARGETS
	gene_sets.gmt#REACTOME_ERK_MAPK_TARGETS
	gene_sets.gmt#KEGG_GRAFT_VERSUS_HOST_DISEASE
	gene_sets.gmt#REACTOME_NUCLEAR_EVENTS_KINASE_AND_TRANSCRIPTION_FACTOR_ACTIVATION
	gene_sets.gmt#PID_AURORA_B_PATHWAY
	gene_sets.gmt#REACTOME_SYNTHESIS_SECRETION_AND_INACTIVATION_OF_GLP1
	gene_sets.gmt#REACTOME_INCRETIN_SYNTHESIS_SECRETION_AND_INACTIVATION
	gene_sets.gmt#PID_PLK1_PATHWAY
**Down**	gene_sets.gmt#KEGG_OXIDATIVE_PHOSPHORYLATION
	gene_sets.gmt#HALLMARK_FATTY_ACID_METABOLISM
	gene_sets.gmt#KEGG_PPAR_SIGNALING_PATHWAY
	gene_sets.gmt#KEGG_PRIMARY_BILE_ACID_BIOSYNTHESIS
	gene_sets.gmt#REACTOME_RESPIRATORY_ELECTRON_TRANSPORT_ATP_SYNTHESIS_BY_CHEMIOSMOTIC_COUPLING_AND_HEAT_PRODUCTION_BY_UNCOUPLING_PROTEINS_
	gene_sets.gmt#REACTOME_SYNTHESIS_OF_BILE_ACIDS_AND_BILE_SALTS
	gene_sets.gmt#REACTOME_RESPIRATORY_ELECTRON_TRANSPORT
	gene_sets.gmt#REACTOME_TRIGLYCERIDE_BIOSYNTHESIS
	gene_sets.gmt#PID_SMAD2_3PATHWAY
	gene_sets.gmt#HALLMARK_ANGIOGENESIS
	gene_sets.gmt#HALLMARK_OXIDATIVE_PHOSPHORYLATION
	gene_sets.gmt#REACTOME_LATENT_INFECTION_OF_HOMO_SAPIENS_WITH_MYCOBACTERIUM_TUBERCULOSIS

Supplementary Table S4: List of primer used for quantitative real-time PCR

Primer name	Accession No.	Nucleotide Sequence (5'→3')	5' Position	Target length
**Sult2a4-F**	NM_001101534.1	TCCAGGGTCACTCAGAACTT	423	72
**Sult2a4-R**	NM_001101534.1	TGCTCAAACCATGATCCATA	494	72
**Ap3b2-F**	NM_021492.3	TTCAAGAAGGAACAGGGAAA	3249	70
**Ap3b2-R**	NM_021492.3	AGGTGTCTGGCAATGTAAGC	3318	70
**Duoxa1-F**	NM_145395.2	CTGGTAGCCACTGAGTTTCC	1312	70
**Duoxa1-R**	NM_145395.2	GGACAGTTCTGCTGGTTTTG	1381	70
**Pnoc-F**	NM_010932.2	GCCCCATCTTCTCACTCATC	1170	70
**Pnoc-R**	NM_010932.2	CCCAGGTCTGATTTCATGTT	1239	70
**Sub1-F**	ENSMUST00000110504	CGGACAGCGAAGTTGAAAAA	164	70
**Sub1-R**	ENSMUST00000110504	TGCTTCTTCACGGGCTTCTC	233	70
**Sult2a1-F**	NM_001111296.2	CCAGGGTCACTCGGAACTTA	481	70
**Sult2a1-R**	NM_001111296.2	GCTCAAACCATGATCCGAAT	550	70
**Sult2a6-F**	NM_001081325.2	GAACGTTCACCCTGGCTAGA	254	70
**Sult2a6-R**	NM_001081325.2	TGAGTCGTGGTCCTTCCTTA	323	70
**GAPDH-F**	NM_008084.3	GGCATTGCTCTCAATGACAA	1132	95
**GAPDH-R**	NM_008084.3	ATGRAGGCCATGAGGTCCAC	1226	95

F, forward; R, reverse

Supplementary Figures

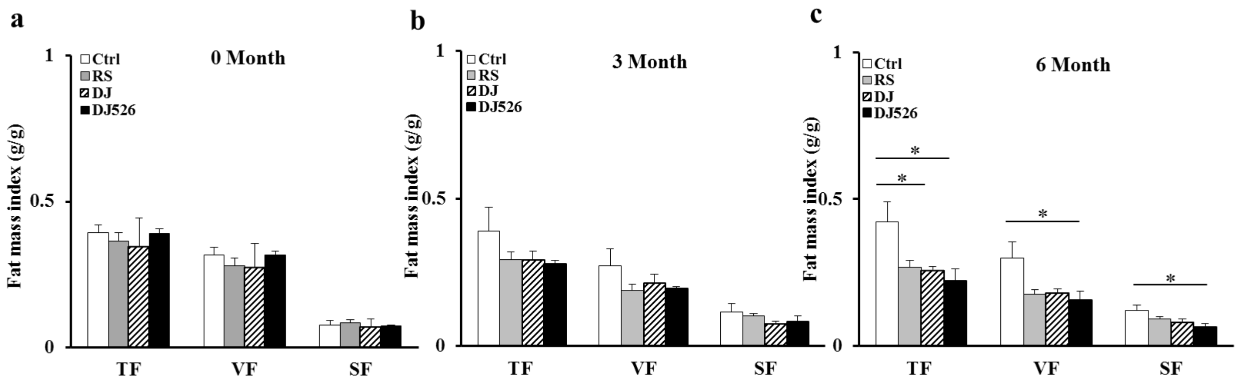

Supplementary Figure S1. The effects of the resveratrol-enriched rice DJ526 on changes in fat mass index with age-progression. The control (Ctrl) mice fed a NFD in which the carbohydrate source was corn starch and sucrose; the resveratrol (RS) mice fed a NFD in which the carbohydrate source was corn starch and sucrose except containing resveratrol; Dongjin (DJ) mice fed a NFD in which the corn starch and sucrose were replaced with Dongjin rice; DJ526, mice fed a NFD in which the corn starch and sucrose were replaced with the resveratrol enriched rice DJ526. The resveratrol concentration in DJ526 and RS groups was 0.69 μg/g each, and not detected in Ctrl and DJ groups. Comparative analysis of the fat mass index (fat mass/total body weight) of mice at 0 month (a), 3 months (b), and at 6 months (c). The values represent the mean ± s.d. (n=20). Values in the figure with a superscripted letter indicate statistical significance as analyzed by an unpaired Student's t-test; *$p < 0.05$ compared with Ctrl.

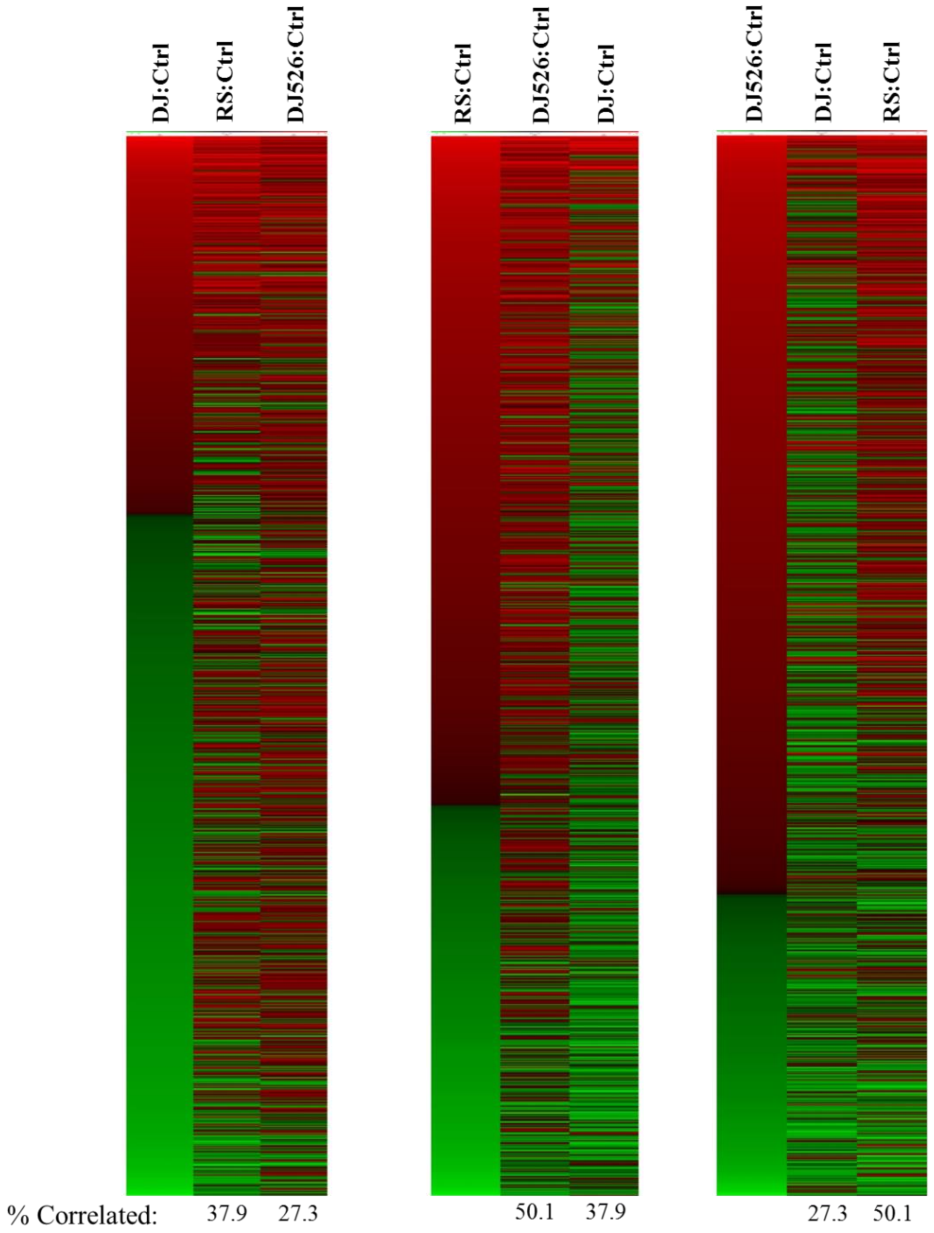

Supplementary Figure S2. The gene set enrichment analysis of the microarray data from the mice on resveratrol (RS), Dongjin rice (DJ), resveratrol-enriched rice DJ526 (DJ526), or control (Ctrl) diet. The control (Ctrl) mice fed a NFD in which the carbohydrate source was corn starch and sucrose; The resveratrol (RS) mice fed a NFD in which the carbohydrate source was corn starch and sucrose except containing resveratrol; Dongjin (DJ) mice fed a NFD in which the corn starch and sucrose were replaced with

Dongjin rice; DJ526, mice fed a NFD in which the corn starch and sucrose were replaced with the resveratrol enriched rice DJ526. The resveratrol concentration in DJ526 and RS groups was 0.69 µg/g each, and not detected in Ctrl and DJ groups. Columns show every pathway significantly up-regulated (red) or down-regulated (green) by each treatment at the end of the 6 month feeding experiments.

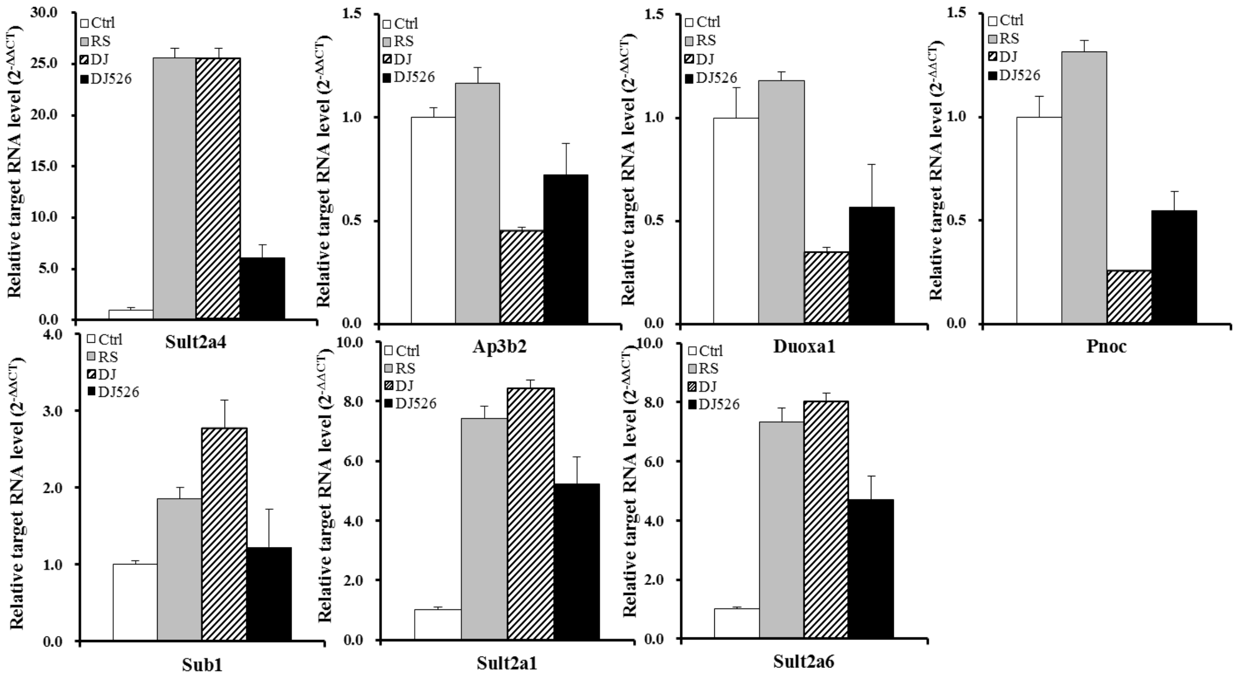

<table>
<tr><th rowspan="2">Gene symbol</th><th colspan="4">Relative target RNA level (2-ΔΔCт)</th><th colspan="3">Z ratio (Array)</th><th colspan="3">Fold change (Array)</th></tr>
<tr><th>Ctrl</th><th>RS</th><th>DJ</th><th>DJ526</th><th>RS/Ctrl</th><th>DJ/Ctrl</th><th>DJ526/Ctrl</th><th>RS/Ctrl</th><th>DJ/Ctrl</th><th>DJ526/Ctrl</th></tr>
<tr><td>Sult2a4</td><td>1</td><td>25.61</td><td>25.52</td><td>6.1</td><td>11.07</td><td>7.99</td><td>6.70</td><td>15.86</td><td>18.48</td><td>10.60</td></tr>
<tr><td>Ap3b2</td><td>1</td><td>1.17</td><td>0.45</td><td>0.72</td><td>5.63</td><td>3.36</td><td>5.13</td><td>4.75</td><td>2.61</td><td>5.15</td></tr>
<tr><td>Duoxa1</td><td>1</td><td>1.18</td><td>0.34</td><td>0.57</td><td>-1.83</td><td>-5.88</td><td>-4.34</td><td>0.87</td><td>0.20</td><td>0.42</td></tr>
<tr><td>Pnoc</td><td>1</td><td>1.31</td><td>0.25</td><td>0.55</td><td>-4.27</td><td>-5.68</td><td>-4.18</td><td>0.49</td><td>0.23</td><td>0.29</td></tr>
<tr><td>Sub1</td><td>1</td><td>1.86</td><td>2.78</td><td>1.22</td><td>3.08</td><td>5.36</td><td>2.17</td><td>2.57</td><td>3.66</td><td>2.09</td></tr>
<tr><td>Sult2a1</td><td>1</td><td>7.44</td><td>8.42</td><td>5.22</td><td>9.13</td><td>6.46</td><td>5.15</td><td>9.49</td><td>10.99</td><td>5.95</td></tr>
<tr><td>Sult2a6</td><td>1</td><td>7.33</td><td>8.02</td><td>4.7</td><td>6.77</td><td>4.63</td><td>3.70</td><td>4.96</td><td>5.00</td><td>3.27</td></tr>
</table>

Supplementary Figure S3. Validation of the microarray results by quantitative real-time PCR from the mice on resveratrol (RS), Dongjin rice (DJ), resveratrol-enriched rice DJ526 (DJ526), or control (Ctrl) diet. Total RNAs from the liver samples of Ctrl, RS, DJ or DJ526 groups were amplified using the primers specific for the indicated targets. The targets were selected based on the Z scores obtained by the microarray analyses, which were provided for the purposes of comparison. Note that Z score numbers reflect statistical confidence and do not relate directly to fold-change.
